# New insights on the photocomplex of *Roseiflexus castenholzii* revealed from comparisons of native and carotenoid-depleted complexes

**DOI:** 10.1016/j.jbc.2023.105057

**Published:** 2023-07-17

**Authors:** Chen-Hui Qi, Guang-Lei Wang, Fang-Fang Wang, Yueyong Xin, Mei-Juan Zou, Michael T. Madigan, Zheng-Yu Wang-Otomo, Fei Ma, Long-Jiang Yu

**Affiliations:** 1 Photosynthesis Research Center, Key Laboratory of Photobiology, Institute of Botany, Chinese Academy of Sciences, Beijing, China; 2 University of Chinese Academy of Sciences, Beijing, China; 3 National Facility for Protein Science in Shanghai, Zhangjiang Lab, Shanghai Advanced Research Institute, Chinese Academy of Science, Shanghai, China; 4 College of Life and Environmental Sciences, Hangzhou Normal University, Hangzhou, China; 5 Department of Microbiology, School of Biological Sciences, Southern Illinois University, Carbondale, Illinois, USA; 6 Faculty of Science, Ibaraki University, Mito, Japan

**Keywords:** photosynthesis, *Roseiflexus castenholzii*, light-harvesting complex, reaction center, core complex, carotenoidless

## Abstract

In wild-type phototrophic organisms, carotenoids (Crts) are primarily packed into specific pigment–protein complexes along with (Bacterio)chlorophylls and play important roles in the photosynthesis. Diphenylamine (DPA) inhibits carotenogenesis but not phototrophic growth of anoxygenic phototrophs and eliminates virtually all Crts from photocomplexes. To investigate the effect of Crts on assembly of the reaction center–light-harvesting (RC–LH) complex from the filamentous anoxygenic phototroph *Roseiflexus* (*Rfl*.) *castenholzii*, we generated carotenoidless (Crt-less) RC–LH complexes by growing cells in the presence of DPA. Here, we present cryo-EM structures of the *Rfl*. *castenholzii* native and Crt-less RC–LH complexes with resolutions of 2.86 Å and 2.85 Å, respectively. From the high-quality map obtained, several important but previously unresolved details in the *Rfl*. *castenholzii* RC–LH structure were determined unambiguously including the assignment and likely function of three small polypeptides, and the content and spatial arrangement of Crts with bacteriochlorophyll molecules. The overall structures of Crt-containing and Crt-less complexes are similar. However, structural comparisons showed that only five Crts remain in complexes from DPA-treated cells and that the subunit X (TMx) flanked on the N-terminal helix of the Cyt-subunit is missing. Based on these results, the function of Crts in the assembly of the *Rfl*. *castenholzii* RC–LH complex and the molecular mechanism of quinone exchange is discussed. These structural details provide a fresh look at the photosynthetic apparatus of an evolutionary ancient phototroph as well as new insights into the importance of Crts for proper assembly and functioning of the RC–LH complex.

In the process of photosynthesis, light energy is absorbed by light-harvesting (LH) complexes and transferred to the reaction center (RC), where charge separation initiates electron transport and subsequent formation of a proton motive force (1). (Bacterio)Chlorophylls ((B)Chls) are the major pigments in LH complexes, whereas carotenoids (Crts) function as accessory absorbers, contributing up to 30% of total harvested light in regions of the spectrum where (B)Chls absorb poorly. This enhanced light-capturing ability facilitates photosynthesis by transferring the absorbed energy to (B)Chls (2, 3). In addition, Crts also play an important photoprotective role by quenching (B)Chl triplet states and harmful oxygen species that enter antenna and RC complexes (4). Crts also play a structural role in stabilizing protein structures (5). Diphenylamine (DPA) is an inhibitor of carotenogenesis and affects the composition and content of Crts in photosynthetic membranes and complexes without inhibiting growth. For example, the Crt content of cells of anoxygenic phototrophic bacteria can be decreased by up to 90% by growing cells in the presence of DPA (6), yielding cells that resemble those of Crt-less mutants (7, 8). DPA-induced Crt-less cells are thus excellent models for investigating the function of Crts in photosynthetic complexes.

*Roseiflexus* (*Rfl*.) *castenholzii* is a thermophilic filamentous anoxygenic phototroph (FAP) of the family *Chloroflexaceae*. Like *Chloroflexus* (*Cfx*.) *aurantiacus*, *Rfl*. *castenholzii* can grow phototrophically under anaerobic conditions in the light or aerobically by respiration in the dark (9). However, unlike *Cfx. aurantiacus, Rfl*. *castenholzii* lacks chlorosomes and contains neither BChl *c* nor carotenes; its major pigments are BChl *a* and γ-carotene derivatives (10), and the composition of the latter can vary depending on culture conditions, such as light intensity, oxygen concentration, and growth phases (11). The RC–LH complex of *Rfl*. *castenholzii* has been characterized by spectroscopic, biochemical, and structural methods (12, 13, 14, 15). However, the absence of a high-resolution structure has been an obstacle to a more thorough understanding of the functioning of this complex. For example, *Rfl*. *castenholzii* contains at least five derivatives of γ-carotene in its RC–LH complex, but only one type of these have been localized in cryo-EM studies of this complex (10, 13, 15).

Here, we report important new details of the *Rfl*. *castenholzii* RC–LH complex revealed from comparisons of the high-resolution cryo-EM structures of native and Crt-less complexes. Based on a high-quality electron density map combined with mass spectrometry analyses, the previously uncertain structural details of the *Rfl*. *castenholzii* RC–LH were determined unambiguously. Three sequences of small polypeptides including subunit X (TMx), one associated with the RC, and one previously identified as the N-terminal helix of the L-subunit, were all clearly assigned, and the functions of two of them were predicted. Moreover, two groups of Crts in the complex were distinguished, and the conformation of B805 molecules (previously designated B800 (15)) was definitively assigned.

Determination of the *Rfl*. *castenholzii* Crt-less RC–LH complex offers the first picture of the assembly of Crts in an RC–LH complex. Our data also reveal the *Rfl*. *castenholzii* RC–LH complex to be structurally unique among quinone-type RC–LH complexes and provide a structural foundation for the future dissection of their energy-transfer mechanisms.

## Results

### Overall structure of the *Rfl. castenholzii* RC–LH complex

**Figure 1:**
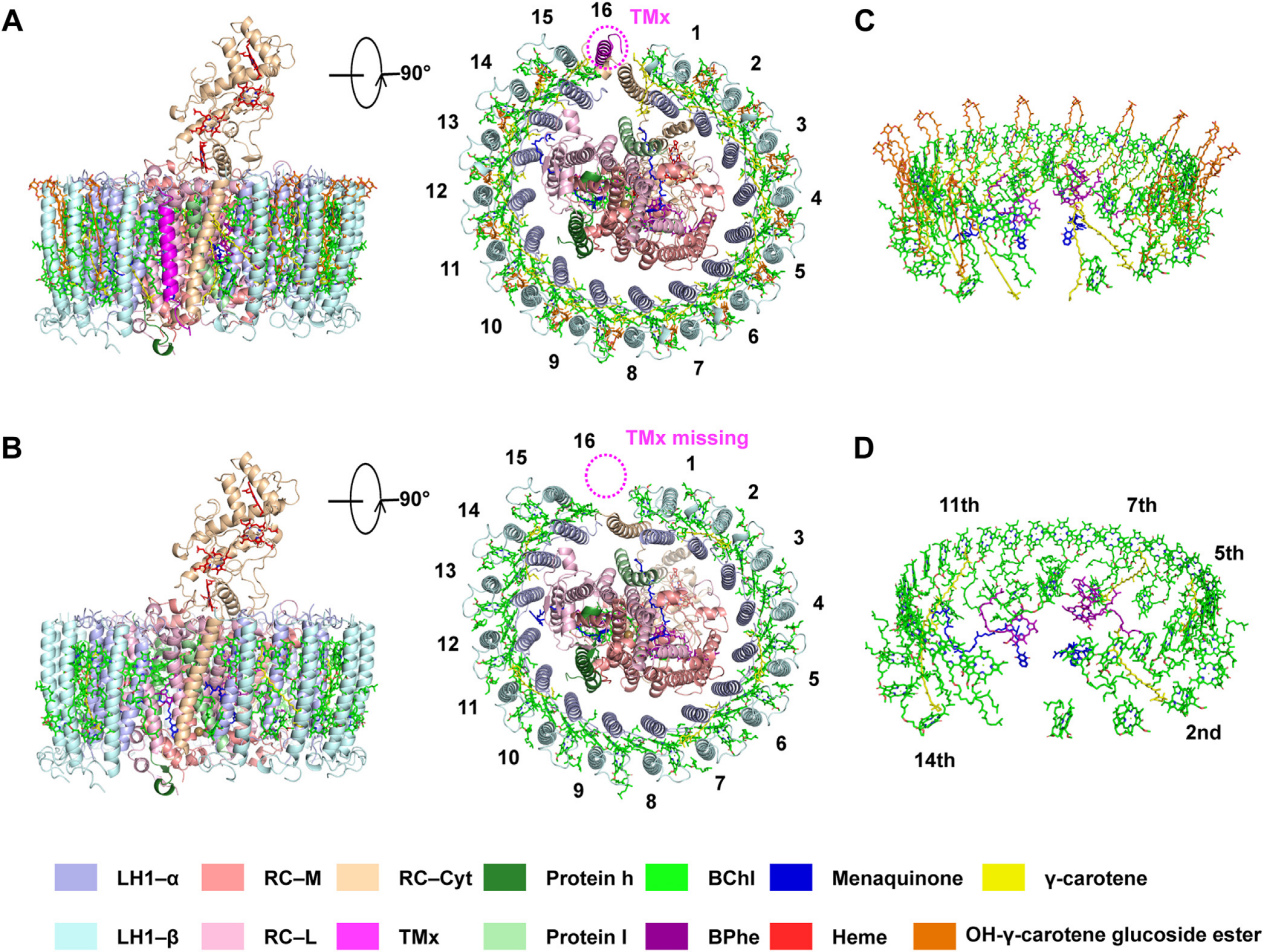
**Overall structure of RC–LH complexes from *Roseiflexus castenholzii*.** Cartoon representations of the native (*A*) and Crt-less (*B*) RC–LH complex are shown at side (*left*) or bottom (*right*) view. Bound pigments of native (*C*) and Crt-less (*D*) RC–LH complex in *stick* representation. *Color* key at the *bottom* of the figure. Crt, carotenoid; LH, light-harvesting; RC, reaction center.

**Figure 2:**
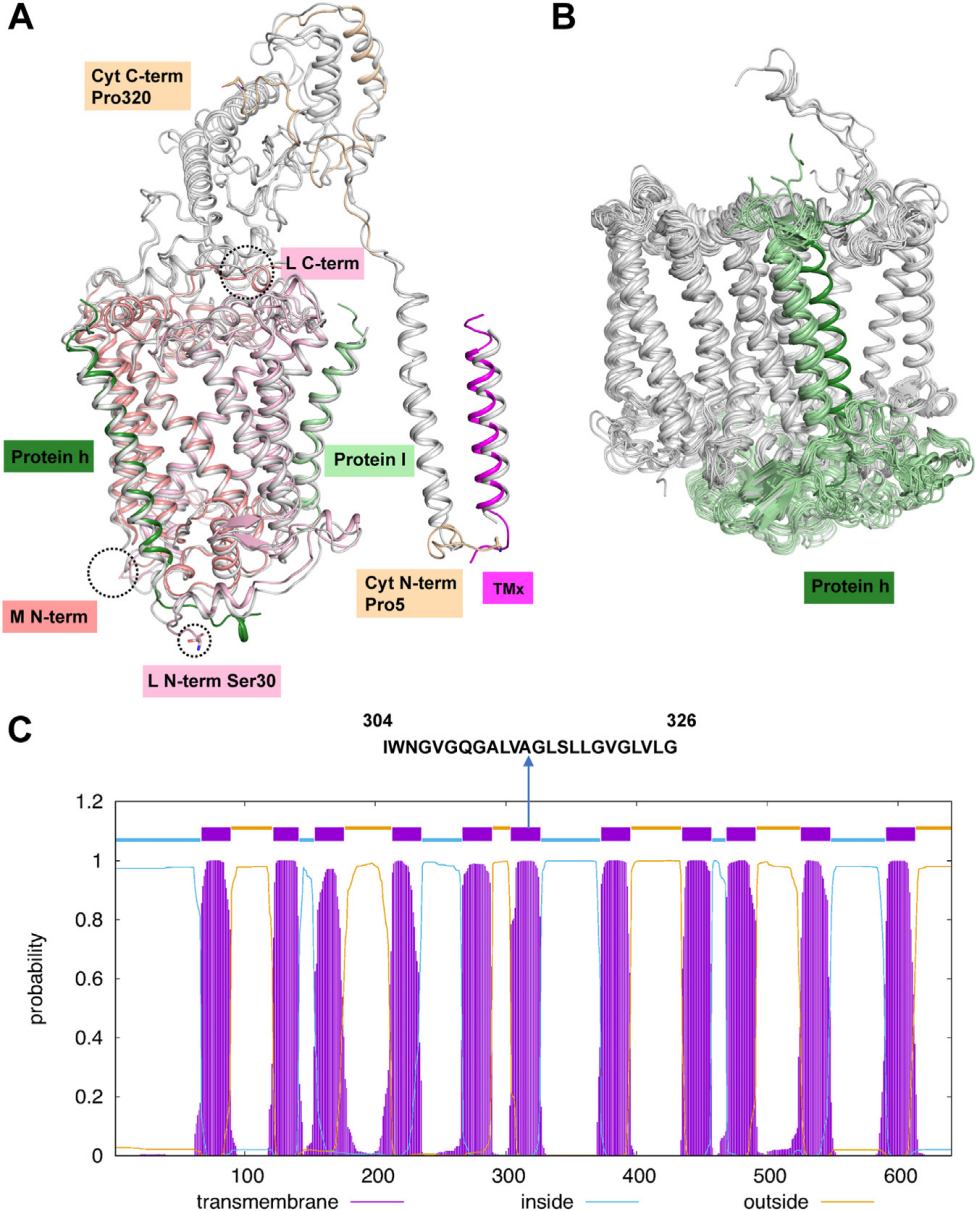
**Structure of *Roseiflexus castenholzii* RC complex.***A*, a comparison with the previously reported structure (Protein Data Bank code: 5YQ7). Regions similar in structure are colored in *gray*, whereas regions with large deviations are colored differently. The C and N terminus of the L- and M-subunits are *circled*, respectively. *B*, the cartoon of purple bacteria RC complexes (overall color is gray except for the H subunit, which is *pale green*; Protein Data Bank codes: *Rhodopseudomonaspalustris*: 6Z5R; *Thermochromatium tepidum*: 5Y5S; *Blastochlorisviridis*: 6ET5; *Trv*. strain 970: 7C9R; *Rhodobacter veldkampii*: 7DDQ; *Rhodospirillum rubrum*: 7EQD; *Rhodobacter sphaeroides*: 7F0L; *Allochromatium tepidum*: 7VRJ; *Rhodopila globiformis*: 7XXF; *Rhodobacter capsulatus*: 7YML), and *Roseiflexus castenholzii* (overall color is *gray* except for protein h: *forest*) were superimposed and cofactors were omitted for clarity. *C*, amino acid sequence of *pufLM* and its 11 consecutive transmembrane regions predicted by the membrane protein topology program TMHMM. The predicted transmembrane region at the end of the L-subunit (the start of the M-subunit) is indicated by an *arrow*. RC, reaction center.

The cryo-EM structure of the native and Crt-less RC–LH complexes of *Rfl. castenholzii* was determined at a resolution of 2.86 Å and 2.85 Å, respectively (Fig. 1, Table S1 and Figs. S1–S6). The general structure of the native complex is in agreement with the previously determined RC–LH complex resolved to 4.1 Å (Protein Data Bank [PDB] code: 5YQ7) (15). However, the r.m.s.d. value between Cα atoms of these two structures is 3.060 Å. This relatively large value is primarily because of deviations from the original assignments of the N-terminal helix of the L-subunit, loop regions in the Cyt-subunit (Fig. 2*A*), and the N termini of the LH β polypeptides; all these were clearly resolved in our high-resolution structure. The *Rfl. castenholzii* RC complex consists of three large subunits (RC-L, RC-M, and RC-Cyt) and locates to the center of the LH ring, and the relatively low r.m.s.d. value observed between the earlier structure and our higher resolution structure was 2.449 Å. Two small transmembrane (TM) polypeptides, renamed protein h and protein I (formerly called proteins TM1 and TM7 (15), respectively), were located near the RC M- and L-subunits (Fig. 2*A*), respectively. Protein h occupies the exact position of the N-terminal helix of the H-subunit in all known structures of purple bacterial RC complexes (16, 17, 18, 19, 20, 21, 22, 23, 24, 25) (Fig. 2*B*), whereas protein I resides close to the irregular region of the LH complex (Fig. 1*A*).

The *Rfl. castenholzii* LH complex consists of 15 pairs of αβ heterodimers forming an open elliptical ring with a gap at the 16th position, which is occupied by the insertion of the N-terminal TM helix of the Cyt-subunit (Cyt-TM) and flanked by the TMx protein on the outside (Fig. 1*A*). The 15 αβ polypeptides bind a total of 45 BChls (30 on the periplasmic side and 15 on the cytoplasmic side) and 30 Crt molecules (16 γ-carotenes and 14 OH-γ-carotene glucoside esters). Of the latter, 15 γ-carotenes are embedded in the TM region between each αβ polypeptide, whereas one locates between the Cyt-TM and the neighboring α polypeptide, and 14 OH-γ-carotene glucoside esters are positioned between two adjacent αβ polypeptides with one OH-γ-carotene glucoside ester missing in the 15th αβ polypeptide; the 16th chimeric pair does not contain BChls or Crts (Fig. 1, *A* and *C*). In addition, lipid and detergent molecules were found in the gap region between the LH and RC complex. Ten lipids containing two fatty-acid chains each along with 16 detergent molecules were found in the complex; however, the precise structure of these lipids could not be determined with confidence.

### A new look at the structure of the *Rfl. castenholzii* RC complex

A unique feature of the *Rfl. castenholzii* RC complex is that its L- and M-subunits are encoded by a fused gene (*pufLM*) rather than by two distinct *puf* genes as is the case in the genomes of all purple bacteria (26). Previous hydrophobicity analysis showed that there are 11 TM helices in the *pufLM* gene product, with six present in the L-subunit and five in the M-subunit (one helix is located near the end of the L-subunit and beginning of the M-subunit, Figs. 2*C* and S7) (26, 27). However, there are insufficient residues to form a TM helix between the L- and M-subunits based on biochemical analyses (15) (Fig. 2*A*). In the original structure, the N terminus of the L-subunit was assigned as TM1 (15), although strangely, it was not predicted to be hydrophobic (Figs. 2*C* and S7). The L-subunit was thus concluded to contain six TM helices, and the independent electron density was assigned as TM7 (15). By contrast, in our higher-resolution structure, the L- and M-subunits are clearly shown to be independent entities containing five TM helices each, and that the N terminus of the L-subunit starts from Ser30 (Fig. 2*A*). Meanwhile, there is a small bulk of electron density about 10 residues near the N terminus of the L-subunit; however, it could not be identified according to the map except for a proline (Fig. S4). Moreover, for the RC Cyt-subunit, our high-quality electron density map showed that the N and C termini extend to Pro5 and Pro320, respectively, and that some loop regions differ from those shown in the original structure (15) (Fig. 2*A*).

Finally, it is noteworthy that the metal atom positioned between the RC Q_A_ and Q_B_ sites was clearly identified by inductively coupled plasma optical emission spectrometer (ICP–OES) in our work to be a Mn ion (Table S2) rather than the Fe found in virtually all known RC–LH1 complexes (16, 17, 18, 19, 20, 21, 22, 23, 24, 25, 28). Compared with the nearly universal presence of Fe in the quinone sites of purple bacterial complexes, the presence of Mn is rare and has been found only in the RC–LH complexes of *Rfl. castenholzii* (this work) and *Cfx. aurantiacus* (29).

### The small polypeptides in the *Rfl*. *castenholzii* RC–LH complex

**Figure 3:**
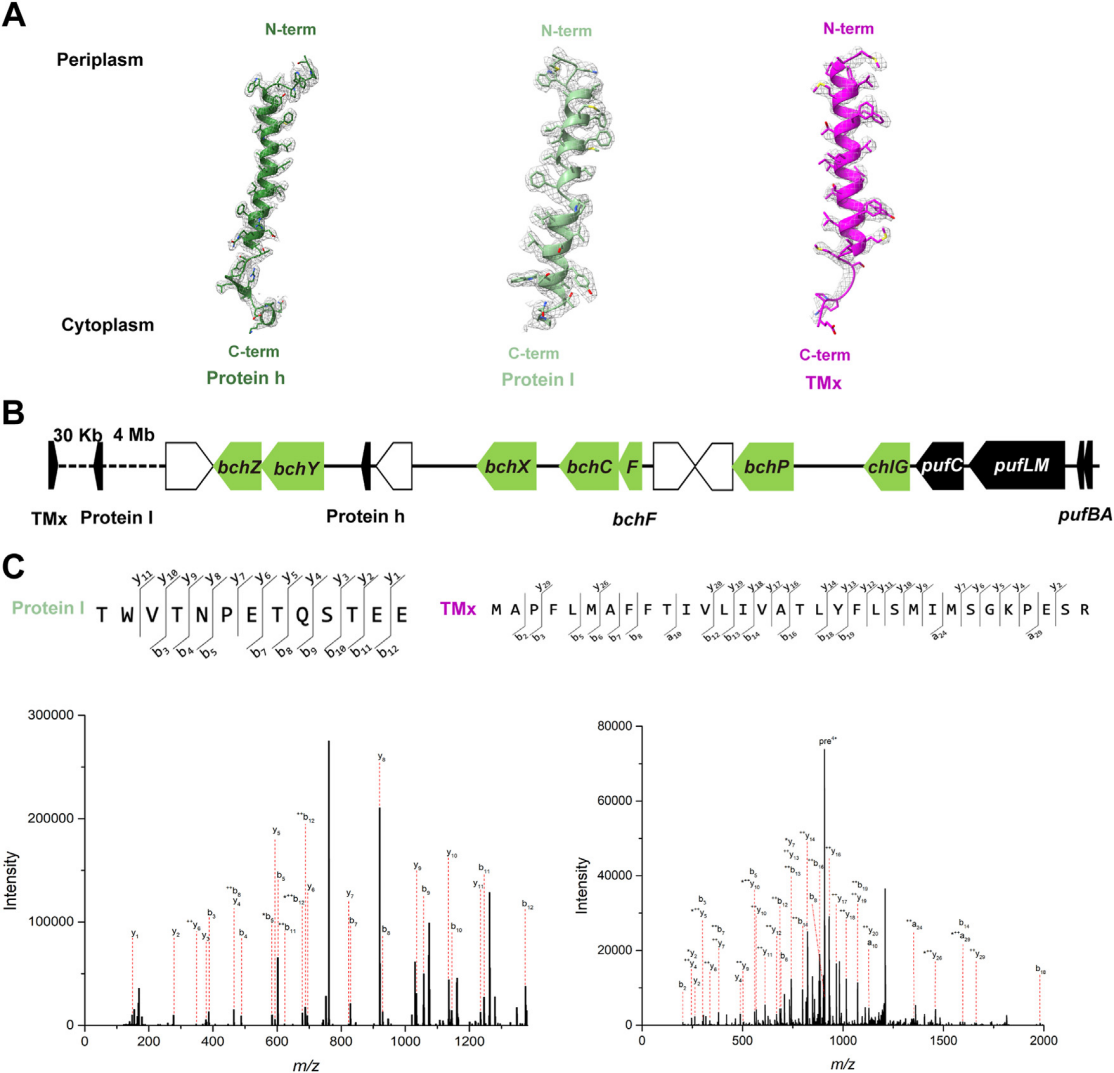
**Identification of three small polypeptides in the RC–LH complex of *Roseiflexus castenholzii*.***A*, cryo-EM densities and structural models of three previously unrecognized small polypeptides. *B*, arrangement of genes in the photosynthetic gene cluster (PGC) and three small polypeptides in *Rfl. castenholzii*. Genes are represented by *rectangles* pointing in the direction of transcription. Genes for bacteriochlorophyll (*bch*) biosynthesis are shown in *green*. The *puf* genes encoding the RC–LH complex and three small polypeptides are shown in *black*. Open reading frames without an assigned gene name were functionally unannotated proteins. *C*, MS/MS fragmentations of TMx and protein I peptides. LH, light-harvesting; RC, reaction center.

In the original structure of the *Rfl*. *castenholzii* RC–LH complex, three small TM polypeptides were identified. Two of these (proteins TMx and TM7) were not characterized, whereas the other (protein TM1) was assigned as the N terminus of the L-subunit (15). By contrast, in our detailed electron density map of this complex, most aromatic residues of TMx were traced unambiguously (Fig. 3*A*). The resulting amino acid sequence could not be accessed from the annotated protein database; however, the gene encoding this polypeptide was identified in the *Rfl. castenholzii* DSM 13941^T^ genome and the protein confirmed by LC–MS/MS (Fig. 3*C*). The gene encoding TMx is located adjacent to genes in the *Rfl*. *castenholzii* genome encoding three iron–sulfur proteins, 1.5 kb upstream of the gene encoding a heterodisulfide reductase–related iron–sulfur protein (WP_012119356.1); this contrasts with the previously assigned sequence of protein TMx to that of a hypothetical protein (WP_041331144.1) (15). Moreover, in our structure, protein TMx is positioned in the complex in an opposite orientation to its previously determined position (15) and to that of other LH αβ polypeptides, with its N and C terminus on the periplasmic and cytoplasmic sides, respectively (Fig. 3*A*).

Two small polypeptides located near the RC were clearly identified in our structure and renamed proteins h and I, respectively (Fig. 3*A*). It was found that TM1 is not the N terminus of the L-subunit as previously described (15), and thus, this protein was renamed protein h because of its similar location to the H-subunit TM helix in all known purple bacterial RCs (16, 17, 18, 19, 20, 21, 22, 23, 24, 25) (Fig. 2*B*). Although the genome of *Rfl. castenholzii* does not contain *puhA* encoding an H-subunit, the presence of protein h in the complex at the precise position of the N-terminal helix of an H-subunit strongly suggests that this helix plays an essential role in RC assembly in those species that contain an H-subunit. The gene encoding protein h is located 10 kb downstream of the *Rfl*. *castenholzii pufBALMC* operon (Fig. 3*B*) and thus may function in a similar manner to that of the H-subunit. The remaining small protein, TM7, was considerably separated from the L- and M-subunits, and no sequence was assigned to it in the previous structure (15). However, employing the same approach as for the TMx sequence, TM7 was positively identified herein and renamed as protein I (Fig. 3, *A* and *C*). The gene encoding protein I is located 30 kb downstream of that encoding TMx (Fig. 3*B*), but the function of protein I is at present unknown.

### The *Rfl. castenholzii* LH αβ heterodimer

**Figure 4:**
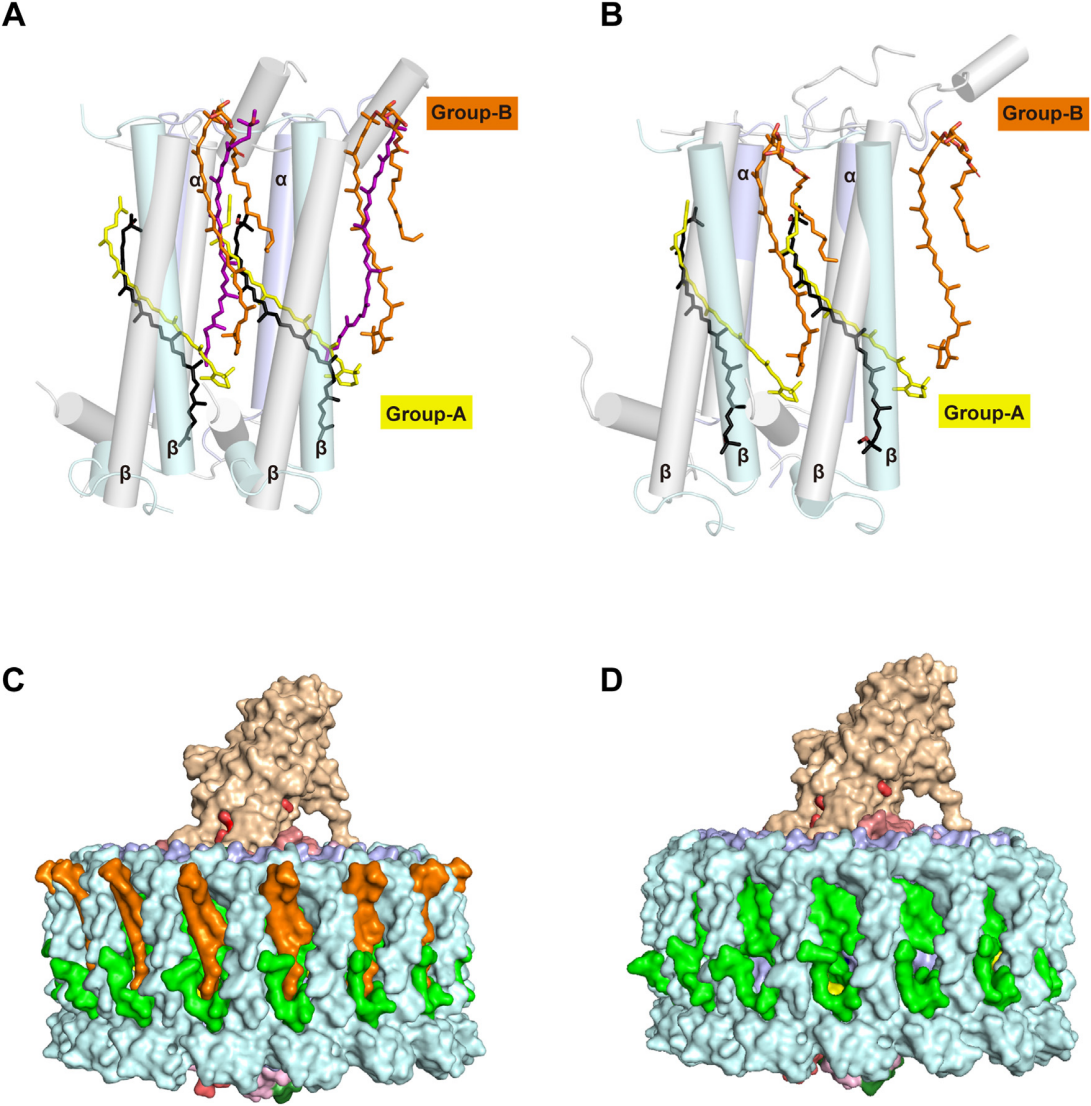
**Carotenoids (Crts) inthe*Roseiflexus castenholzii* LH complex.***A* and *B*, γ-carotene (*yellow sticks* for group A) and OH-γ-carotene glucoside ester (*orange sticks* for group B) in the *Rfl. castenholzii* native LH in comparison with the Crts in the LH1 from (*A*) *Rba. sphaeroides* (*black sticks* for group A, *purple sticks* for group B; Protein Data Bank code:7F0L) and (*B*) *Tch. tepidum* (*black sticks* for group A; Protein Data Bank code: 5Y5S). LH1 αβ-polypeptides between *Rfl. castenholzii* were respectively transparent *light blue* and *pale cyan* cylinders and LH1 αβ polypeptides between *Rba. sphaeroides* and *Tch. tepidum* were transparent *gray cylinders*. *C* and *D*, side view of the surface representation shows a sealed fence for the *Rfl. castenholzii* native (*C*) and Crt-less (*D*) RC–LH complex. γ-carotene (group A) and OH-γ-carotene glucoside ester (group B) were colored *yellow* and *orange*, respectively. LH, light-harvesting; RC, reaction center.

**Figure 5:**
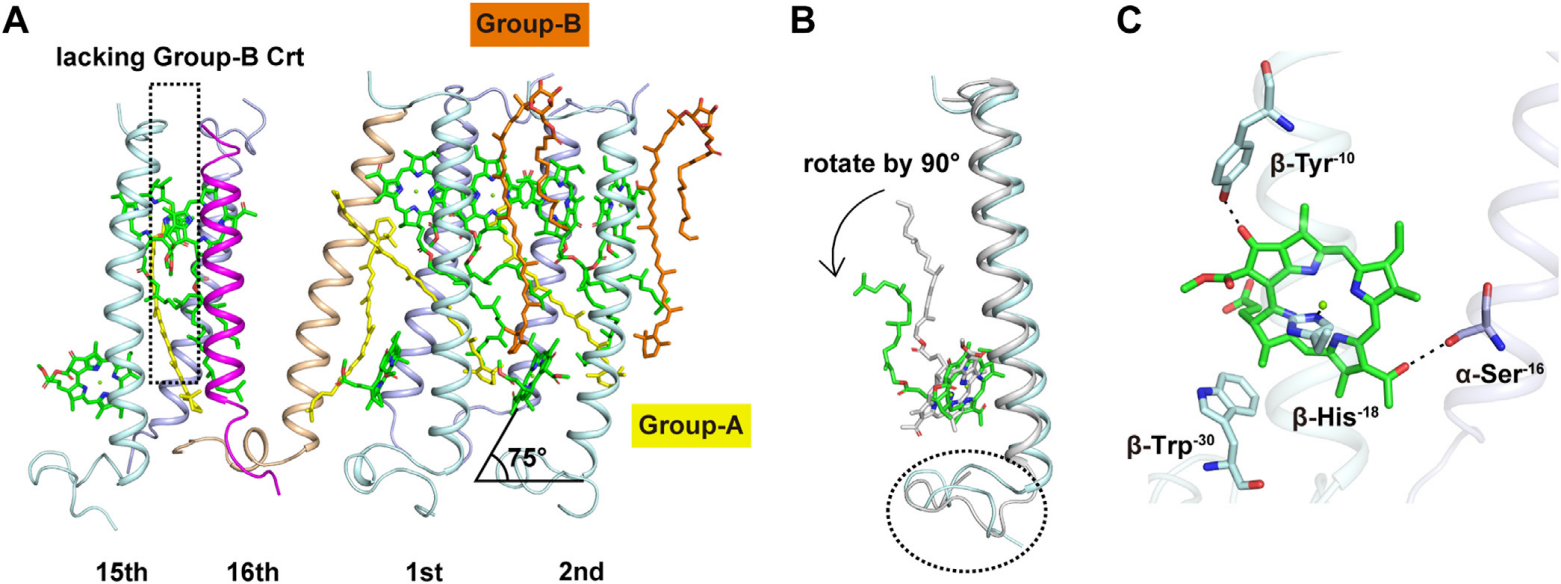
**The irregular region of the LH ring of *Roseiflexus castenholzii*.***A*, the first, second, and 15th αβ polypeptides with the coordinated pigments and auxiliary 16th heterodimer are shown. The lack of the OH-γ-carotene glucoside ester (group B) between the 15th and 16th αβ polypeptides is marked with a *box*. *Color* coding as for Figure 1. *B*, comparison of B805-bound β-polypeptide between the previously determined (Protein Data Bank code: 5YQ7, *gray*) and present structure in our study (*pale cyan*). The large deviation of the N-terminal region is marked with a *circle*. *C*, close view of the B805 coordination in the LH complex. LH, light-harvesting.

In our structure, a total of 30 all-*trans* Crts were clearly resolved in the *Rfl. castenholzii* LH (Fig. 1*C*), corresponding to a ratio of approximately two Crts per αβ heterodimer. This is similar to that in the purple bacteria *Rhodobacter* (*Rba*.) *sphaeroides* (22, 30, 31, 32, 33) (Fig. 4*A*) and *Rba. capsulatus* (34) but differs from most other LH complexes, where each αβ heterodimer contains only one Crt (16, 17, 19, 21) (Fig. 4*B*). The *Rfl. castenholzii* Crts could be divided into two groups, A and B, based on their biochemistry, orientation, and position in the LH complex (Figs. 4 and S4). Group A Crts are deeply embedded in the TM region between the α- and β-polypeptides and have all their β-rings pointing toward the cytoplasmic side (Fig. 5*A*), a position similar to those in most other LH1 complexes; a comparison of the Crt conformations in *Rfl. castenholzii* and the purple bacterium *Thermochromatium* (*Tch*.) *tepidum* is shown in Figure 4*B*. Biochemical analyses have shown that *Rfl*. *castenholzii* produces a mixture of different carotene family Crts (10, 13). Our HPLC analyses of the *Rfl. castenholzii* LH found that the γ-carotene content was about 18% and the OH-γ-carotene glucoside ester about 58% (Fig. S8) that are in agreement with Crt analyses of *Cfx. aurantiacus* (11, 35). Based on the electron density map, we designated group A Crts as γ-carotene (Fig. S4).

In contrast to group A Crts, group B Crts are positioned between two adjacent αβ heterodimers (instead of inside the αβ heterodimer as in group A Crts), which is similar to that of group B Crts from *Rba. sphaeroides* (22, 30, 31, 32, 33) (Fig. 4*A*) and *Rba. capsulatus* (34). The *Rfl. castenholzii* group B Crts were identified as OH-γ-carotene glucoside ester esterified with C_14_ saturated fatty acids (Fig. S4), with the β-ring facing the cytoplasmic side and the glucopyranosyl group facing the periplasmic side of the membrane (Fig. 4*A*). The β-ring of each group A Crt is positioned near the bacteriochlorin ring of the B805 BChl *a* at the N terminus of the n + 1 polypeptide, whereas the Crt tail is near the bacteriochlorin ring of the B880 BChl *a* at the C terminus of the n–1 polypeptide. This arrangement brings the β-ring of a group B Crt close to the β-ring of group A Crt and bacteriochlorin ring of B805 and brings a portion of the group B Crt located on the periplasmic side close to the bacteriochlorin ring of B880 (Fig. 5*A*). This allows the two Crts to effectively interact with the n + 1, n, and n–1 BChls. Collectively, the group B OH-γ-carotene glucoside esters combined with the group A γ-carotenes fill the space between the adjacent αβ heterodimers (Fig. 4*C*) resulting in a blockage of the pores observed in the LH1 complexes of phototrophs containing only group A Crts (17).

### The *Rfl. castenholzii* “LH2-like” structure

Another interesting feature of photosynthesis in *Rfl. castenholzii* is that the organism has an “LH2-like” LH complex, which means that the LH complex contains two groups of BChl *a* molecules, B880 and B805. In the previously published *Rfl. castenholzii* RC–LH structure (15), B880 was properly assigned; however, we have found that the bacteriochlorin ring of B805 should be rotated by 90° as shown in Figure 5*B*. This B805 conformation is important because it reveals two previously unrecognized hydrogen bonds between the B805 C3-acetyl and α-Ser^-16^ and the B805 C13^1^-carbonyl and β-Tyr^-10^ (Figs. 5*C* and S9, *A* and *B*). These bonds allow each B805 molecule to link each pair of αβ heterodimers. The adjacent Mg–Mg distance and the Q_*y*_ transition resemble those of B800 in LH2, but the conformation of B805 is distinct from that in LH2. The dihedral angle between the B805 bacteriochlorin ring plane and the membrane plane is 75° in the *Rfl. castenholzii* complex (Fig. 5*A*), whereas the B800 bacteriochlorin ring plane in purple bacterial LH2s is almost parallel to the membrane (36, 37). In addition, the N-terminal loop regions of *Rfl. castenholzii* LH β polypeptides revealed in our high-resolution structure were found to have an opposite spanning direction compared with the previous structure (15) (Fig. 5*B*), although the position of β-Trp^-30^ remained the same and is thus unable to interact with B805 (Fig. 5*C*).

### Comparison of the native and Crt-less *Rfl. castenholzii* RC–LH complexes

The addition of DPA did not affect the growth of phototrophic cultures of *Rfl. castenholzii* (Fig. S10*B*) but significantly suppressed the Crt content with a gradual decrease observed as cells were serially subcultured (Fig. S10*A*). By the ninth subculture, the Crt content reached a minimum as assessed by absorption spectroscopy (Fig. S10*A*). Thus, the RC–LH complex in cells from this culture was isolated and purified for structure determination.

The overall structure of the DPA-induced Crt-less RC–LH complex was similar to the native complex (r.m.s.d. values of 0.866) except that it contained only five Crt molecules per complex and did not contain the TMx protein (Fig. 1, *B* and *D*). Twenty-seven lipids and 16 detergent molecules were detected, but their precise nature could not be identified. The space occupied by group B Crts in the native complex was filled by lipids containing two fatty-acid chains in the Crt-less complex, and the total lipid content of the latter was much higher.

In the Crt-less *Rfl. castenholzii* LH, five group A Crts were positioned near the second, fifth, seventh, 11th, and 14th αβ polypeptides (Fig. 1, *B* and *D*). Their structures were nearly unchanged compared with their native counterparts (Figs. 1*B* and S1–S6). However, the absence in the Crt-less complex of all group B and most group A Crts had three major consequences. First, the pores in the LH complex that block quinone transport in the native LH were open (Fig. 4, *C* and *D*). Second, the rigidity of the LH complex was compromised because of the missing structural contribution of Crts and the TMx protein, which is consistent with the result that a close interaction between Crts and LH1 proteins plays a key role in enhancing the thermal stability of *Blastochloris tepida* RC–LH1 complexes (38). And third, the Q_*y*_ transitions of B880 and B805 molecules were affected, which could be seen as a blue shift of Q_*y*_ absorption from 880 to 878 nm for B880 and from 805 to 800 nm for B805 (Fig. S1*A*). The average Mg-to-Mg distances between B880 and B805 in the two complexes differed only slightly, and as a result, a change in BChl exciton coupling could not be fully responsible for the observed blue shift. A more likely explanation is that the absence of a Crt–BChl coupling interaction decreased the energy of the excitonic BChl. In addition, since TMx was absent from the Crt-less LH, the Cyt-TM is slightly shifted (Fig. 1*B*).

## Discussion and conclusion

Previous biochemical studies have suggested that the RC complex of *Rfl. castenholzii* consists of L-, M-, and Cyt-subunits only (26). *Cfx. aurantiacus* also lacks the RC H-subunit; so it is believed that this is a common feature of FAPs (39, 40, 41). However, our high-resolution structure of the *Rfl. castenholzii* RC–LH revealed that the position of the N-terminal helix of the purple bacterial H-subunit is occupied in the *Rfl. castenholzii* RC–LH complex by protein h (Fig. 2*B*), indicating that protein h may function in a manner similar to that of the H-subunit and explain the absence of *puhA* in FAPs. In early studies, *puhA* deletion mutants were used to examine the effect of Crts on the formation of LH1 and the RC in the purple bacterium *Rhodospirillum* (*Rsp*.) *rubrum*, and the results indicated that the H-subunit is unessential for formation of LH1 and photosynthetic membranes but essential for the assembly of a functional RC (42). This suggests that protein h may be essential for the assembly of a functional RC–LH in *Rfl. castenholzii*. A gene encoding a hypothetical protein (sequence ID: NWG19514.1) with 91.49% identity to protein h has also been found in metagenomic analyses of *Chloroflexus*-like hot spring bacteria (Fig. S9*C*), suggesting that a protein h-like TM helix may exist in the RC–LH of other *Chloroflexi*.

The RC–LH complex of *Rfl. castenholzii* shows some of the common features of purple bacteria, in particular its LH2-like LH complex. The two-ring organization of BChls incorporates B880 (as in the purple bacterial LH1 core complex) and B805 (as in the purple bacterial LH2 peripheral complex), indicating that the *Rfl. castenholzii* LH complex may fulfill both core and peripheral LH functions. The *Rfl. castenholzii* B805 coordination is different from that of all known LH2 complexes. Two binding modes are known for the B800 of LH2 in purple bacteria. One is the coordination of Mg by the N-terminal α-Met^-30^ whose amino group is modified by carboxylation and its C3-acetyl forms hydrogen bonds with β-Arg^-10^ (Fig. S9, *A* and *B*). *Rba. sphaeroides* and *Rhodoblastus* (*Rbl*.) *acidophilus* use this type of binding (36, 37). The other binding mode is the use of Asp^-28^ near the N terminus of the α polypeptide to bind Mg and β-Thr^-13^ (or β-His^-13^) to bind C3-acetyl (Fig. S9, *A* and *B*), as used by *Phaeospirillum* (*Phs*.) *molischianum* and *Marichromatium* (*Mch*.) *purpuratum* (43, 44). In contrast to both these, B805 in *Rfl. castenholzii* binds in a novel manner with β-His^-18^ binding Mg and α-Ser^-16^ binding C3-acetyl (Fig. 5*C*). Notably, β-His^-18^ is conserved but not involved in B800 binding in all known LH2 complexes, whereas the known B800-binding site in LH2 is absent in the *Rfl. castenholzii* LH (Fig. S9, *A* and *B*). In addition, the planes of B800 of *Rba. sphaeroides* and *Rbl. acidophilus* LH2 are almost parallel to the membrane (36, 37), whereas the planes of B800 in *Phs. molischianum* and *Mch. purpuratum* LH2 are tilted away from the membrane plane by 38° (43, 44). By contrast, the plane of the B805 of *Rfl. castenholzii* is at 75° relative to the membrane (Fig. 5*A*). These three aspects suggest that B805 in *Rfl. castenholzii* is a third class of B805 molecules distinct from the B800 of purple bacterial LH2. Interestingly, β-Tyr^-10^ of the *Rfl. castenholzii* LH is also conserved in *Cfx. aurantiacus* (Fig. S9*B*), which suggests that B805 of *Cfx. aurantiacus* is similar in arrangement to that of *Rfl. castenholzii*.

Each LH subunit of the *Rfl. castenholzii* LH contains two Crts, similar to that of *Rba. sphaeroides* (22, 30, 31, 32, 33) and *Rba. capsulatus* LH1s (34) but different from most LH1s of purple bacteria where only one Crt is present per αβ polypeptide (16, 17, 19, 21). In *Rba. sphaeroides* LH1, the closely spaced Crts were thought to block the quinone exchange channel between adjacent LH1 subunits (22), whereas the large opening in the LH1 ring formed by PufX and protein U (elsewhere named as PufY or protein Y) provided the main channel for quinone exchange (22, 30, 31, 32, 33). By contrast, in the *Rfl. castenholzii* LH, group B combined with group A Crts fill the pores between the adjacent 15 αβ-subunits (Fig. 4*C*), pores that were thought to be channels for quinones to move from the RC to the quinone pool outside the LH complex during photosynthetic electron flow (17, 45). The tightly sealed LH “fence” of *Rfl. castenholzii* is thus likely unpenetrable, and as a result, alternative strategies are needed to facilitate quinone transport. In this connection, we predict that the absence of Crts in the 16th αβ-subunit where Cyt-TM and TMx are located is the pathway for quinone exchange in the native *Rfl. castenholzii* RC–LH complex. In the Crt-less LH complex where most Crts are absent, pores exist between adjacent LH subunits (Fig. 4*D*), and these likely allow for quinone exchange in a manner similar to that of the thermophilic purple bacterium *Tch*. *tepidum* (17). Surprisingly, however, because growth of cells of *Rfl. castenholzii* containing native or Crt-less complexes was virtually the same (Fig. S10*B*), both mechanisms must facilitate quinone exchange at similar rates.

In summary, our study has provided a more detailed look at the structure of the *Rfl. castenholzii* native RC–LH complex and the first look at a corresponding structure from Crt-depleted cells. Comparison of these structures revealed new details of the architecture of this photocomplex, including proteins and cofactors, and the consequences of removing its Crts, including deletion of the TMx protein (now known to be unessential for quinone exchange) and a shift in Cyt-TM position. Collectively, our findings offer a new look at important aspects of photosynthesis in an early branching phototroph including structure–function relationships in light harvesting and energy transfer, mechanisms of quinone transport, and the function of Crts in the assembly of LH complexes.

## Experimental procedures

### Cultivation of *Rfl. castenholzii* cells

*Rfl. castenholzii* cells were grown phototrophically (anoxic/light) at 50 °C for 7 days as described previously (14). The culture was illuminated by a 40-W incandescent lamp. For DPA-induced species, native bacteria were inoculated into liquid media containing 20 mg/l (118 μM) DPA with other growth conditions exactly the same as for native cultivation. DPA-treated cells were then subcultured into the same DPA-containing medium and then subcultured eight more times to obtain cells with the lowest Crt content.

### Preparation of native and Crt-less RC–LH complexes

*Rfl. castenholzii* native and Crt-less cells were harvested by centrifugation, resuspended in 20 mM Tris–HCl buffer (pH 7.5), and broken by sonication (Ultrasonic Homogenizer JY92-IIN; SCENTZ). The suspensions were centrifuged at 27,216*g* for 15 min (Avanti J-26S XPI Centrifuge; BECKMAN COULTER) to remove unbroken cells and debris, and the supernatant was subjected to ultracentrifugation at 208,429*g* for 2 h (OptimaTM L-100 XP Ultracentrifuge; BECKMAN COULTER). The chromatophores obtained were solubilized with 0.85% (w/v) *n*-octyl-β-d-glucoside at room temperature for 1 h and ultracentrifuged again for 3 h. The chromatophores obtained were solubilized with 0.85% (w/v) *n*-dodecyl β-d-maltopyranoside at room temperature for 30 min and ultracentrifuged for 1 h to obtain RC–LH-rich fractions. These were loaded onto a di-ethyl-amino-ethyl anion-exchange column (Toyopearl 650S; TOSOH), equilibrated with 20 mM Tris–HCl (pH 7.5) containing 0.05% (w/v) *n*-dodecyl β-d-maltopyranoside, and eluted with a linear gradient of 50 to 175 mM NaCl. An RC–LH fraction with A880/A280 >1.5 was collected, concentrated with polyethylene glycol 1450 to a final protein concentration of 13.5% (w/v), and resuspended in 20 mM Tris–HCl buffer (pH 7.5) for subsequent studies.

### Cryo-EM data collection

Two microliters of the protein solution were applied on glow-discharged holey carbon grids (200 mesh Quantifoil R2/2 molybdenum) that had been treated with H_2_ and O_2_ mixtures in a Solarus plasma cleaner (Gatan) for 30 s and then blotted and plunged into liquid ethane at −182 °C using an EM GP2 plunger (Leica, Microsystems). The applied parameters were a blotting time of 6 s at 80% humidity and 4 °C. Data were collected on a Talos Arctica (Thermo Fisher Scientific) electron microscope at 300 kV equipped with a K3 camera (Gatan). Movies were recorded using EPU software (Thermo Fisher Scientific) at a nominal magnification of 81,000× in super-resolution mode (yielding a pixel size of 0.89 Å after two times of binning) for native RC–LH complex and in counting mode (yielding a pixel size of 0.89 Å) for Crt-less RC–LH complex. Each frame was exposed for 0.067 s, and the total exposure time was 2.67 s, leading to a total accumulated dose of 58 e^−^/Å^2^ for the native RC–LH complex and of 56 e^−^/Å^2^ for the Crt-less RC–LH complex.

### Image processing of the native RC–LH complex

Cryo-EM data were processed by CryoSPARC except where specified elsewhere (46). A total of 9428 collected movies were subject to patch motion correction (F-crop factor = 1/2) and patch CTF estimation, yielding micrographs at 0.89 Å/pixel with CTF parameters; crYOLO (Institute of Molecular Physiology, Group Raunser, Thorsten Wagner) was used to perform automated particle picking (47). A pretrained model was applied to pick 1,773,448 particles at a threshold of 0.1 with a box size of 200. Picked particles were subjected to two rounds of 2D classification. About 1,241,745 good particles were selected for further process. Manually curated exposures were applied to remove micrographs whose CTF fit a resolution lower than six and relative ice thickness exceeding 1.06. A total of 1,089,075 particles from high-quality micrographs were subject to two rounds of 2D classification from which 826,289 particles from 52 classes were selected and re-extracted at 360 pixels for 3D reconstruction and 791,877 particles were extracted. Four models were reconstructed by a CryoSPARC built-in *ab initio* reconstruction tool followed by heterogeneous refinement. The best class with 639,374 particles was selected for treatment of two rounds of nonuniform refinement job with CTF refinement (48, 49). For the first refinement, the inner window radius was set to 0.6, yielding a density map at 2.91 Å resolution, whose output was used for a final refinement with inner window radius equal to 0.85, yielding a final map at 2.86 Å resolution according to the gold-standard Fourier shell correlation using a criterion of 0.143. Local resolution maps were calculated on CryoSPARC's built-in local resolution estimation tool.

### Image processing of Crt-less RC–LH complex

Cryo-EM data were processed by CryoSPARC except where specified elsewhere (46). All stacked movies were subject to patch motion correction, and defocus was estimated by patch CTF estimation. A total of 1,316,239 particles were autopicked by crYOLO with the pretrained model applied in which the picking threshold was set to 0.1 and the box size set to 200 (47). After two rounds of 2D classification, 425,223 good particles were sorted out for 3D reconstruction. Three initial models were produced by *ab initio* reconstruction followed by heterogeneous refinement. Two classes containing 373,675 particles were selected to perform nonuniform refinement (48, 49). For the first refinement, the inner window radius was set to 0.6, yielding a density map at 2.89 Å resolution. A second refinement was performed with input from the first refinement, and CTF refinement was applied, producing a final map with a resolution of 2.85 Å with a Fourier shell correlation threshold at 0.143.

### Model building and refinement of the RC–LH complex

The 4.1 Å atomic model of the *Rfl. castenholzii* RC–LH (PDB code: 5YQ7) (15) was fitted into the cryo-EM density map of *Rfl. castenholzii* RC–LH using Chimera (Computer Graphics Laboratory, Department of Pharmaceutical Chemistry, University of California) (50). Real space refinement for the peptides and cofactors were performed using COOT (York Structural Biology Laboratory, University of York) (51). TMx and protein h were modeled *ab initio* based on the density map. The manually modified model was real space refined on PHENIX (Molecular Biophysics and Integrated Bioimaging Division, Lawrence Berkeley National Laboratory, Berkeley) (52, 53), and the COOT/PHENIX refinement was iterated until the refinements converged. Finally, statistics calculated by PHENIX were checked, and figures were drawn with the PyMOL Molecular Graphic System (Schrödinger) (54), UCSF Chimera (50), and UCSF ChimeraX (55).

### ICP–OES and absorption spectroscopy measurements

Metals were determined by measuring the Mn and Fe ratios in *Rfl. castenholzii* RC–LH complexes having an absorbance of 125 at 880 nm using an ICP–OES (56) (Thermo iCAP 6300). Absorption spectra of RC–LH complexes were collected using a UV-1900i UV–Vis spectrophotometer (SHIMADZU) scanning at 0.5 nm intervals between 250 and 1000 nm.

### LC–MS/MS analysis

*Rfl. castenholzii* RC–LH complexes were subjected to SDS-PAGE gel electrophoresis. Gels containing proteins under 40 kDa were cut into small pieces and destained in a 25 mM ammonium bicarbonate/50% acetonitrile buffer. Proteins were reduced with 10 mM DTT in 50 mM ammonium bicarbonate at 56 °C for 1 h, alkylated with 55 mM iodoacetamide in 50 mM ammonium bicarbonate in the dark for 45 min, and then digested with trypsin overnight at 37 °C. Peptides in the gel were extracted by two rounds of ultrasound in a buffer containing 5% trifluoroacetic acid and 50% acetonitrile. The liquid was freeze-dried with a SpeedVac, and the peptides were desalted using a StageTip. For MS analysis, peptides were resuspended in 0.1% (v/v) formic acid (FA) and analyzed by an Orbitrap Fusion Lumos Tribrid mass spectrometer (Thermo Fisher Scientific) coupled online to an Easy-nLC 1200 in a data-dependent mode. The peptides were separated by reversed-phase LC on a 150 μm (inner diameter) × 250 mm (length) analytical column packed with 1.9 μm diameter C18 particles. For each cycle, a complete MS scan was obtained in an Orbitrap at 120 K resolution with an automatic gain control (AGC) target of 5 × 10^5^ followed by MS/MS for 3 s for the most intense precursors. Higher-energy collisional dissociation was used to fragment these precursors at a normalized higher-energy collisional dissociation collision energy of 32%, and these fragments were analyzed in Orbitrap.

For top–down analysis, 1 mg/ml of *Rfl. castenholzii* RC–LH complex was desalted with Zip Tip C4 and dissolved in mobile phase A (0.1% FA in ddH_2_O), which was manually sampled into a reverse-phase C4 column (filled with 3 μm particle size C4 material in a 25 cm length column with 150 μm internal diameter, Dr Maisch GmhH, Inc), connected to an Easy nLC-1200 system, and then analyzed by LC–MS/MS using an Orbitrap Fusion Lumos liquid mass spectrometer. The samples were eluted with 30 to 100% mobile phase B (0.1% FA and 20% water in acetonitrile) for 30 min and continued with 100% B for 40 min at a flow rate of 600 nl/min. The MS1 parameters of the Orbitrap Fusion Lumos mass spectrometer were set to an Orbitrap resolution of 120 K, a scan range of 350 to 2000 *m/z*, an AGC target of 500,000, and an include charge state of 3 to 20 s. In MS2, the activation type was electron-transfer dissociation. Supplemental activation collision energy was set to 15%, Orbitrap resolution was at 120 K, and the AGC target was 300,000 for analysis. The experiment was repeated three times for each condition.

The entire protein sequence of *Rfl. castenholzii* from the National Center for Biotechnology Information and the two protein sequences of protein I and TMx obtained in the translated genome were used to form a new database. The MS data obtained by trypsin digestion of the *Rfl. castenholzii* RC–LH complex and top–down analysis were converted to Mascot generic format. Searches were performed in the new database using Mascot generic format. Mascot 2.5.1.3 (Matrix Science) uses a peptide tolerance of 10 ppm and an MS/MS tolerance of 0.6 Da, and the b-y ion series were searched. Peptides obtained from the search were considered to be correctly identified when the Mascot score was >20 and the expectation value was <0.05.

### Extraction and HPLC analysis of Crts

Pigments of the *Rfl. castenholzii* native RC–LH complex were extracted with acetone–methanol (7:2, v/v), and the extracts separated by HPLC on a C18 column (TSKgel Super-ODS, 4.6 mm × 10 cm, particle size 2.3 μm) eluted with methanol (1.0 ml/min) at wavelengths of 190 to 800 nm with detection wavelengths of 770 and 475 nm used for BChl *a* and Crts, respectively.

## Data availability

The cryo-EM density maps were deposited in the Electron Microscopy Data Bank (www.ebi.ac.uk/pdbe/emdb/) under the following accession codes: EMD-35721 for native RC–LH complex and EMD-35727 for Crt-less RC–LH complex. The atomic coordinates have been deposited in the PDB (www.rcsb.org) under the following accession codes: 8IUG and 8IUN. All other data are available from the corresponding authors upon reasonable request.

## Supporting information

This article contains supporting information.

## Conflict of interest

The authors declare that they have no conflicts of interest with the contents of this article.
