# Supplementary material for: New insights on the photocomplex of Roseiflexus castenholzii revealed from comparisons of native and carotenoid-depleted complexes
Source: J Biol Chem. 2023 Jul 17;299(8):105057. doi: 10.1016/j.jbc.2023.105057 (PMC10432797; doi:10.1016/j.jbc.2023.105057)
Supplement: Supporting Figures S1–S10 and Tables S1 and S2 [file mmc1.pdf]

Supplementary Materials for

New insights on the photocomplex of *Roseiflexus castenholzii* revealed from comparisons of native and carotenoid-depleted complexes

Authors: Chen-Hui Qi, et al.

***Corresponding author. Email: fma@ibcas.ac.cn; longer@ibcas.ac.cn.**

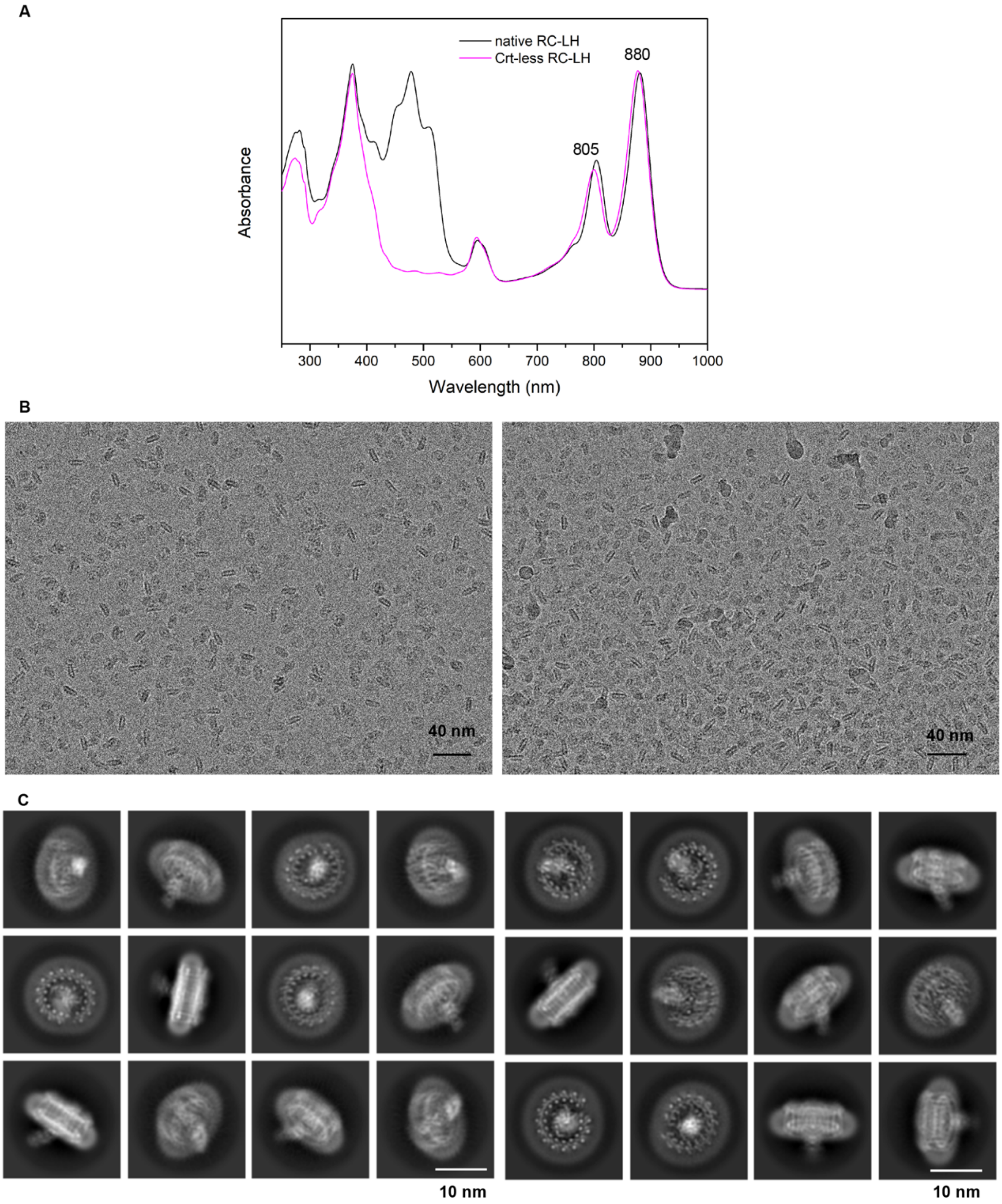

Fig. S1 Absorption spectrum and cryo-EM of the *Rfl. castenholzii* native and Crt-less RC–LH complexes. (A) Absorption spectrum of the purified native (dark) and Crt-less (magenta) RC–LH complexes at room temperature. **(B)** Representative cryo-EM micrographs of native (left) and Crt-less (right) RC–LH complexes. **(C)** Representative 2D class averages processed from the micrographs of native (left) and Crt-less (right) RC–LH complexes.

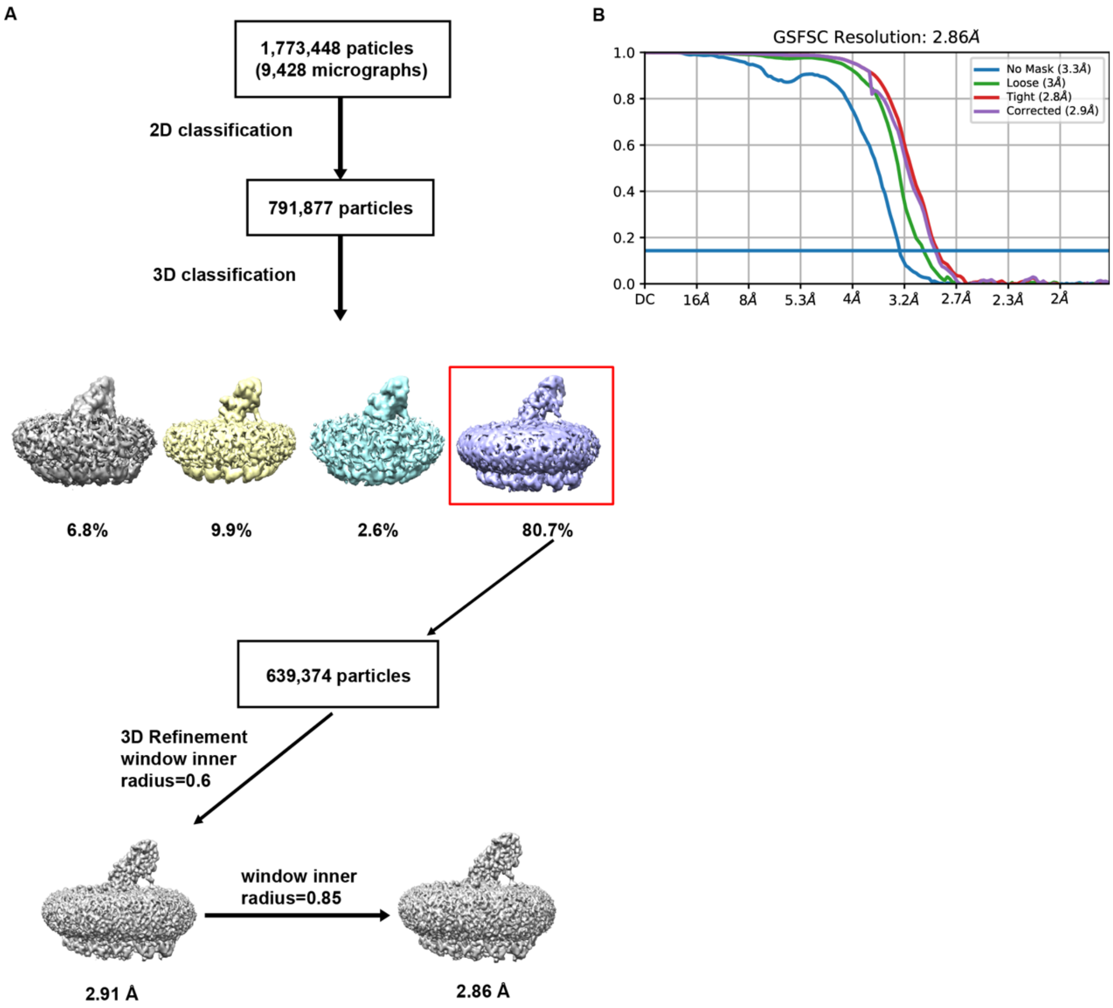

Fig. S2 Structure determination of the *Rfl. castenholzii* native RC–LH complex. (A) Image processing flow of 3D classification and reconstruction. **(B)** The Fourier shell correlation (FSC) plot of the cryo-EM map (No mask: blue, spherical: orange, loose: green, tight: red, corrected: purple).

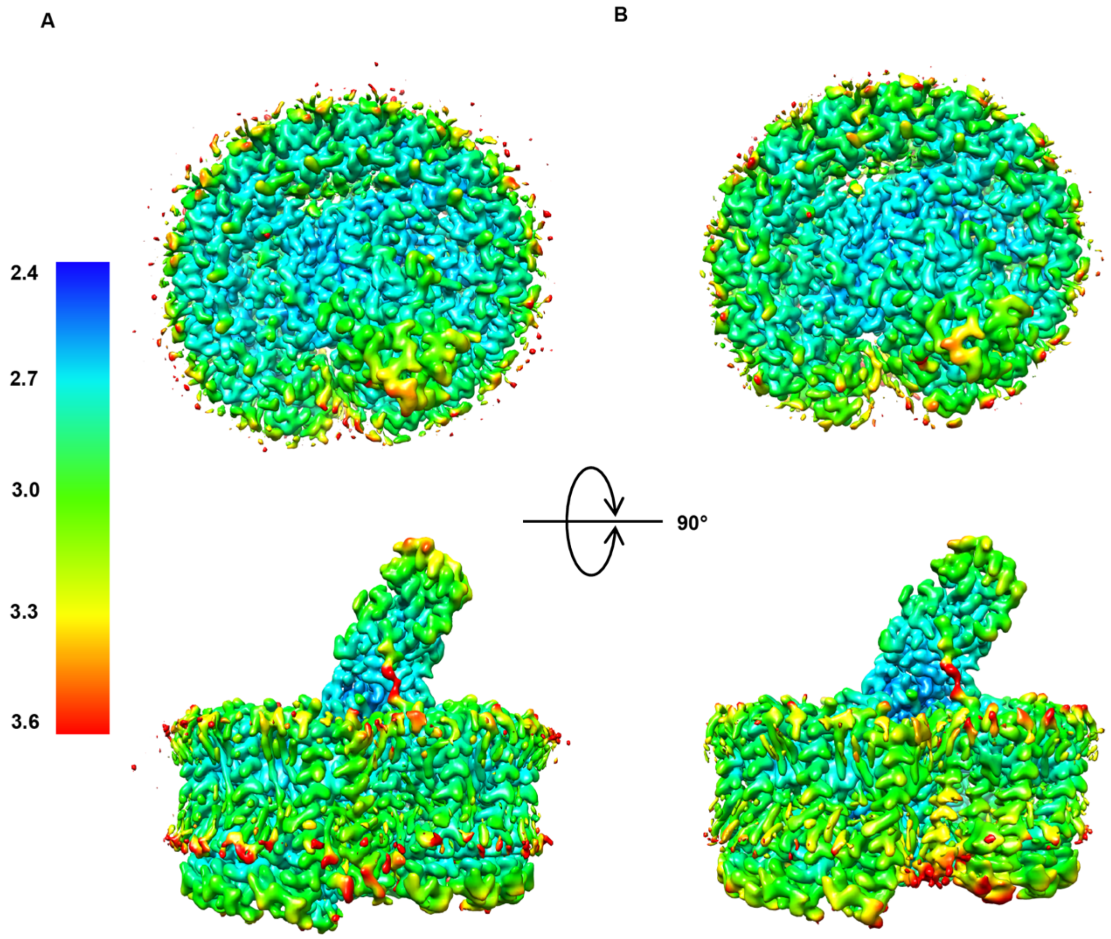

Fig. S3 Local resolution representation of the structure of RC–LH complex. Top view from periplasmic side (up) and side view (down) parallel to the membrane plane of native **(A)** and Crt-less **(B)** RC–LH complexes. The map is shown in the colors of the rainbow according to the estimated resolution from 3.6 Å (red) to 2.4 Å (blue).

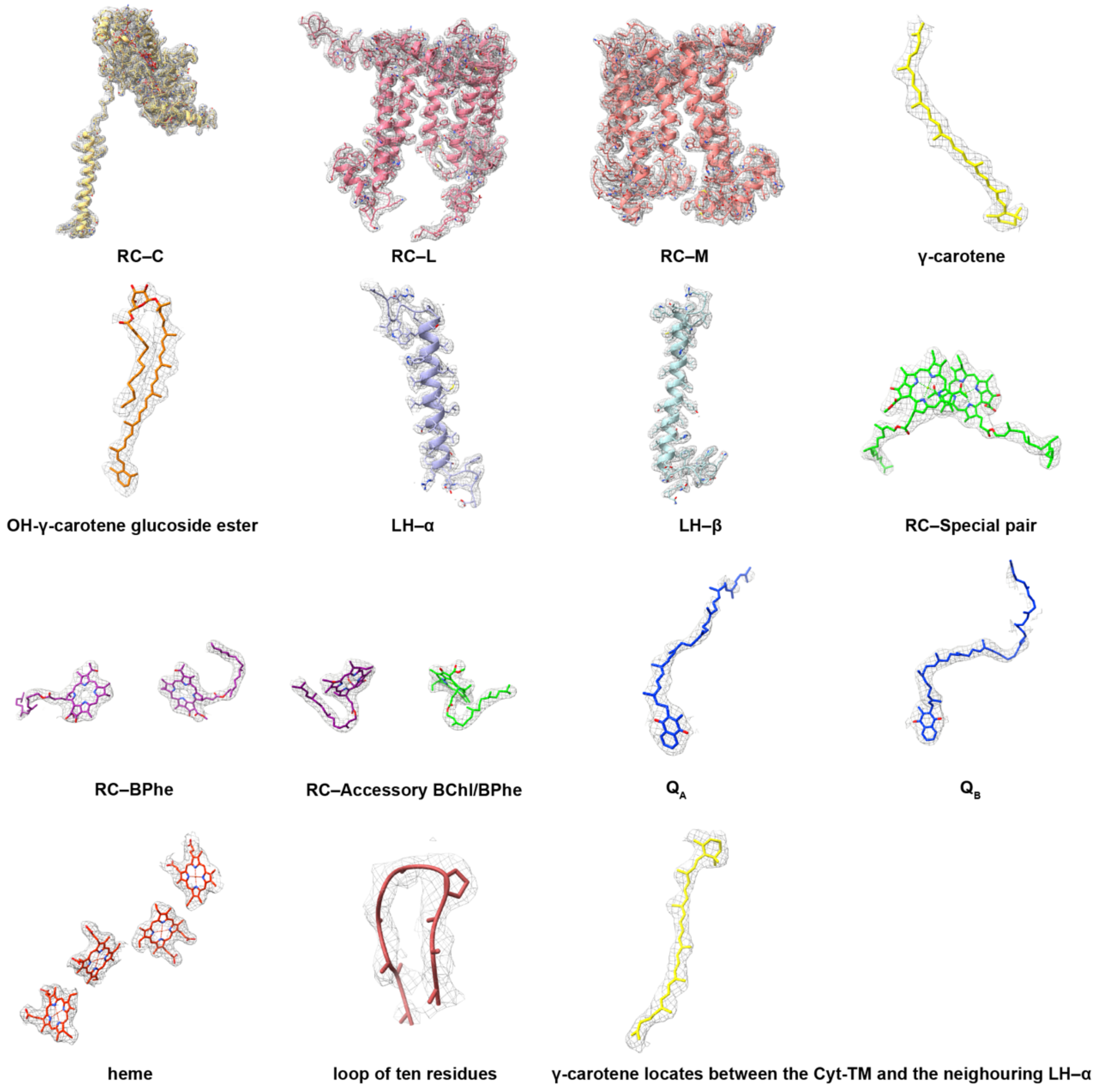

Fig. S4 Cryo-EM densities and structural models in the *Rfl. castenholzii* native RC–LH complex. The color codes of polypeptides are the same as in Fig. 1.

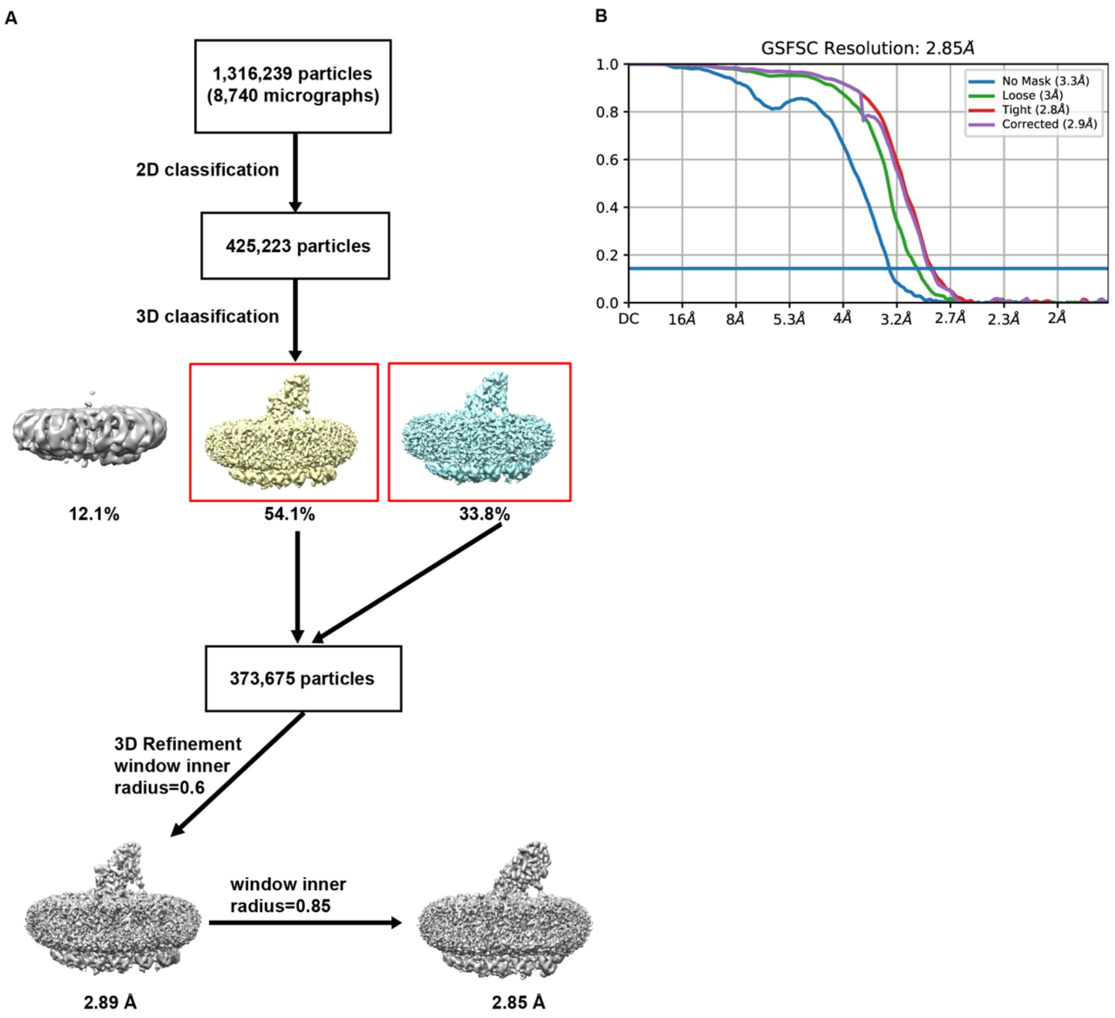

Fig. S5 Structure determination of the *Rfl. castenholzii* Crt-less RC–LH complex. **(A)** Image processing flow of 3D classification and reconstruction. **(B)** The FSC plot of the cryo-EM map (No mask: blue, spherical: orange, loose: green, tight: red, corrected: purple).

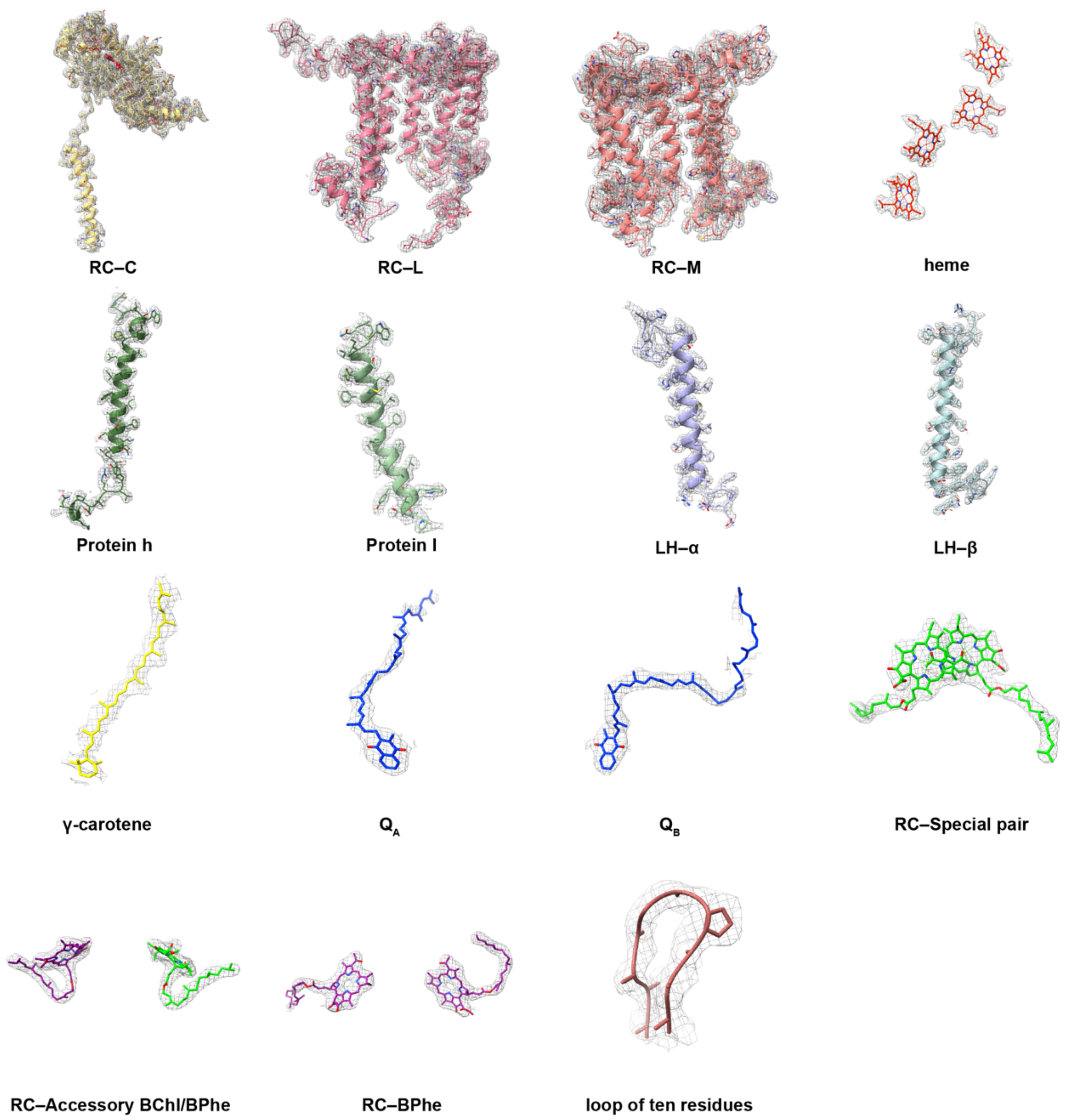

Fig. S6 Cryo-EM densities and structural models in the *Rfl. castenholzii* Crt-less RC–LH complex. The color codes of polypeptides are the same as in Fig. 1.

MSAVPRALPL PSGETLPAEA ISSTGSQAAS AEVIPFSIIE EFYKRPGKTL AARFFGVDPF DFWIGRFYVG LFGAISIIGI ILGVAFYLYE GVVNEGTLNI

L-TM1

LAMRIEPPPV SQGLNVDPAQ PGFFWFLTMV AATIAFVGWL LRQIDISLKL DMGMEVPIAF GAVVSSWITL QWLRPIAMGA WGHGFPLGIT HHLDWVSNIG

L-TM2 L-TM3

YQYYNFFYNP FHAIGITLLF ASTLFLHMHG SAVLSEAKRN ISDQNIHVFW RNILGYSIGE IGIHRVAFWT GAASVLFSNL CIFLSGTFVK DWNAFWGFWD

L-TM4 L-TM5

KMPIWNGVGQ GALVAGLSLL GVGLVLGRGR ETPGPIDLHD EEYRDGLEGT IAKPPGHVGW MQRLLGEGQV GPIYVGLWGV ISFITFFASA FIILVDYGRQ

TM6 M-TM1

VGWNPIIYLR EFWNLAVYPP PTEYGLSWNV PWDKGGAWLA ATFFLHISVL TWWARLYTRA KATGVGTQLA WGFASALSLY FVIYLFHPLA LGNWSAAPGH

M-TM2 M-TM3

GFRAILDWTN YVSIHWGNFY YNPFHMLSIF FLLGSTLLLA MHGATIVATS KWKSEMEFTE MMAEGPGTQR AQLFWRWVMG WNANSYNIHI WAWWFAAFTA

M-TM4 M-TM5

ITGAIGLFLS GTLVPDWYAW GETAKIVAPW PNPDWAQYVF R

M-TM5

Fig. S7 Amino acid sequence of *pufLM*. The transmembrane helices of L- and M-subunits are marked with blue wavy lines in present structure, whereas gray indicates amino acids not resolved according the cryo-EM density map. The 6th transmembrane helix of pufLM predicted by TMHMM program is marked by the red wavy line.

A

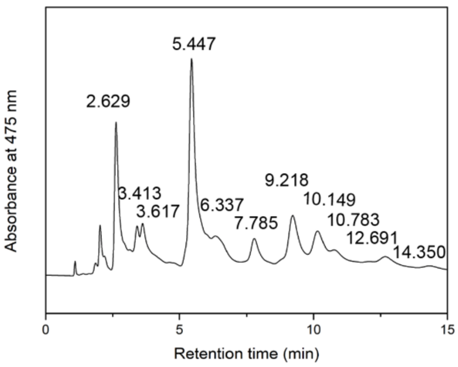

B

Composition	Retention time (min)	Content (%)
keto-OH-γ-carotene	2.629	16.13
methxy-keto-γ-carotene	3.413	2.38
keto-γ-carotene	3.617	3.97
OH-γ-carotene glucoside ester	5.447	42.46
	6.337	8.29
	7.785	5.07
γ-carotene	9.218	10.17
	10.149	6.35
	10.783	2.16
OH-γ-carotene glucoside ester	12.691	2.10
	14.350	0.93

C

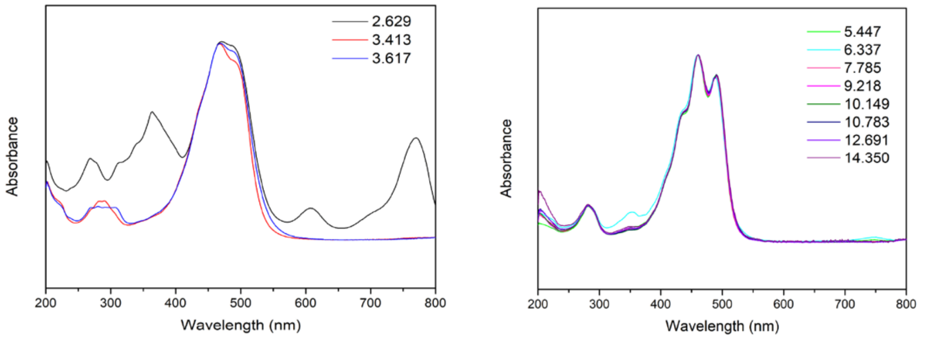

Fig. S8 Pigments analysis of RC–LH complex of *Rfl. castenholzii.* (A) HPLC chromatogram of the pigment extract from the native RC–LH complex. **(B)** Content of corresponding elution bands. **(C)** Absorption spectra of the corresponding elution bands.

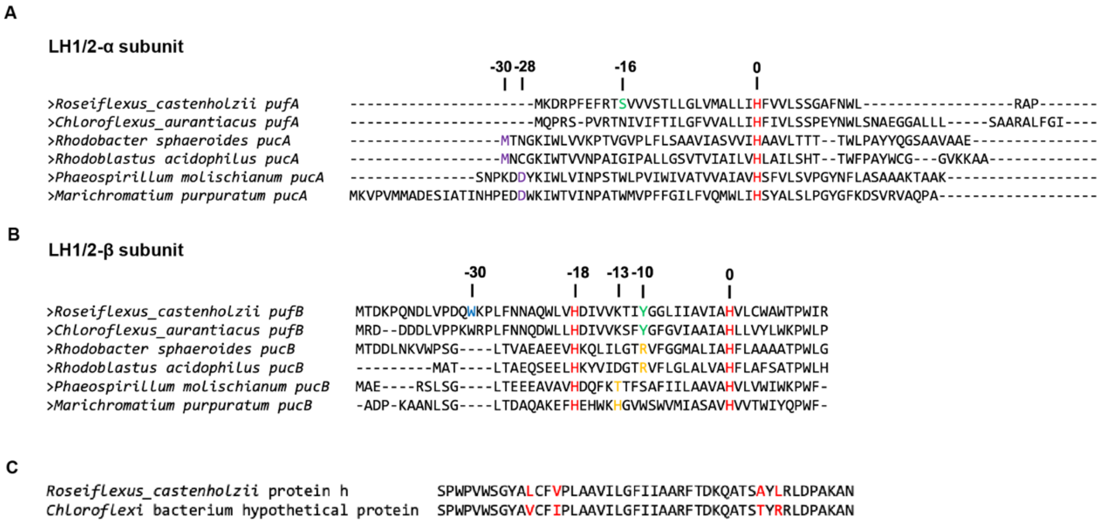

Fig. S9 Sequence alignments of α/β-polypeptides and protein h for typical anoxygenic phototrophs. (A) Sequence alignments of the LH1 α-polypeptides of *Rfl. castenholzii* and *Cfx. aurantiacus* and LH2 α polypeptides of other purple bacteria. **(B)** Comparison of the LH1 β-polypeptides of *Rfl. castenholzii* and *Cfx. aurantiacus* and LH2 β polypeptides of other purple bacteria. **(C)** Comparison of *Rfl. castenholzii* protein h and *Chloroflexi* bacterium hypothetical protein (Sequence ID: NWG19514.1).

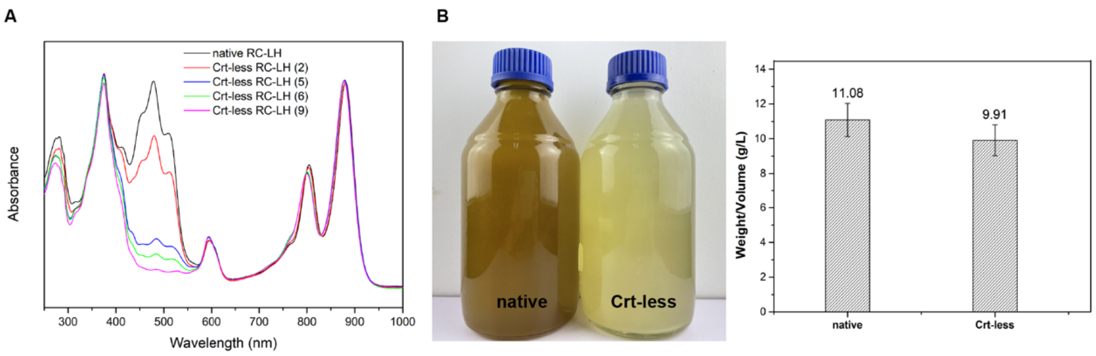

Fig. S10 Characterizations of Crt-less *Rfl. castenholzii* RC–LH complex. (A) The RC–LH absorption spectra of different subcultures after the addition of Diphenylamine (DPA). **(B)** Comparison of native and Crt-less *Rfl. castenholzii* growth in light.

Table S1 Cryo-EM data collection, refinement and validation statistics.

	native RC–LH complex (EMD-35721, PDB ID: 8IUG)	Crt-less RC–LH complex (EMD-35727, PDB ID: 8IUN)
**Data collection and processing**		
Magnification	81000 ×	81000 ×
Voltage (kV)	300	300
Electron exposure (e–/Å^2)	58	56
Defocus range (μm)	-0.8 to -1.6	-0.8 to -1.6
Pixel size (Å)	0.89	0.89
Symmetry imposed	C1	C1
Initial particle images (no.)	1773448	1316239
Final particle images (no.)	639374	373675
Map resolution (Å)	2.86	2.85
FSC threshold	0.143	0.143
**Refinement**		
Initial model used (PDB code)	5YQ7	8IUG
Model resolution (Å)	4.1	2.86
FSC threshold	0.143	0.143
Map sharpening *B* factor (Å^2)	105.5	96.5
Model composition		
Non-hydrogen atoms	25238	24278
Protein residues	2356	2318
Ligands	133	128
B factors (Å^2)		
Protein	48.34	33.98
Ligand	47.59	38.66
R.m.s. deviations		
Bond lengths (Å)	0.012	0.015
Bond angles (°)	2.054	2.075
Validation		
MolProbity score	1.55	1.62
Clashscore	5.53	6.97
Poor rotamers (%)	0.00	0.00
Ramachandran plot		
Favored (%)	96.23	96.39
Allowed (%)	3.77	3.61
Disallowed (%)	0.00	0.00

Table S2 Metal elements were determined in the RC–LH complex using Inductively Coupled Plasma Optical Emission Spectrometer (ICP–OES)

Metals	Fe/259.9 nm	Mn/257.6 nm
unit	ppm（mg/L）	ppm（mg/L）
average	9.131	2.153
ratio of average	4	1
Standard deviation	0.092	0.025
%RSD	1.005	1.149
Repeat#1	9.066	2.135
Repeat#2	9.196	2.170
